# Mechanisms by which complex carbohydrates influence immune imbalance in COPD via the gut–lung axis: from colonic fermentation to pulmonary immune responses

**DOI:** 10.3389/fnut.2026.1833070

**Published:** 2026-04-28

**Authors:** Guanglei Chen, Yunzhi Chen, Cancan Chu, Xing Zhu

**Affiliations:** School of Basic Medical Sciences of Traditional Chinese Medicine (Qihuang College), Guizhou University of Traditional Chinese Medicine, Guiyang, Guizhou, China

**Keywords:** chronic obstructive pulmonary disease, colonic fermentation, complex carbohydrates, gut–lung axis, immune imbalance, short-chain fatty acids, tryptophan metabolites

## Abstract

Chronic obstructive pulmonary disease (COPD) is characterized not only by local airway inflammation and tissue injury, but also frequently by persistent systemic immune imbalance. After entering the colon, complex carbohydrates can be converted by the gut microbiota into gut-derived molecules such as short-chain fatty acids (SCFAs) and tryptophan metabolites, which may further influence the pulmonary immune status in COPD. These effects are mainly related to the regulation of colonic fermentation kinetics and metabolite production by substrate structure, as well as to the actions of selected metabolites on pulmonary immune cells and airway epithelium after intestinal absorption and systemic distribution. The monosaccharide composition, glycosidic linkage type, degree of branching, and degree of polymerization of complex carbohydrates can affect colonic fermentation kinetics and further alter the production ratio of SCFAs and tryptophan metabolites. SCFAs are the main candidate metabolites linked to the regulation of aberrant neutrophil recruitment, alveolar macrophage inflammatory status, the Treg/Th17 balance, and airway epithelial barrier integrity; selected tryptophan metabolites are mainly involved in mucosal defense and epithelial repair. In COPD, bile acids are more likely to be associated with microaspiration from gastroesophageal reflux and local microecological alterations. Complex carbohydrates may participate in the regulation of immune imbalance in COPD by affecting the production, distribution, and local pulmonary actions of gut-derived metabolites, but the quantitative relationships among these processes across the gut, blood, and lung, as well as their specific pulmonary effects in COPD, still require further clarification, particularly in human studies with synchronized sampling.

## 1 Introduction

Chronic obstructive pulmonary disease (COPD) is commonly characterized by persistent airflow limitation and structural damage to the airways and alveoli (1). Under long-term exposure to cigarette smoke and environmental stimuli, patients exhibit sustained abnormal accumulation of immune cells and secretion of inflammatory mediators in the local airways (2–4). In addition to local airway alterations, patients with COPD often present with increased concentrations of pro-inflammatory factors and abnormal differentiation of immune cells across organs in the peripheral blood. This abnormal immune status in the peripheral circulation is associated with an increased risk of acute exacerbations and the progression of extrapulmonary comorbidities (5, 6). The long-term coexistence of local lesions and peripheral immune abnormalities suggests that COPD progression is driven not only by intrapulmonary inflammation, but also by the persistent influence of cross-organ metabolic and immune signals.

The local immune abnormalities in the airways of COPD are mainly manifested by persistent neutrophil accumulation, reduced phagocytic clearance of pathogens and apoptotic cells by alveolar macrophages, an imbalance in the proportion of regulatory T cells (Treg) and T helper 17 (Th17) cells, as well as abnormal cytokine secretion and barrier function in airway epithelial cells (7–11). These abnormal states jointly promote persistent local inflammation, aggravated tissue degradation, and impaired repair capacity. However, current inhaled therapies mainly act on bronchodilation and local inflammation control, with limited effects on the regulation of persistent immune abnormalities in the peripheral circulation and the cross-organ inflammatory milieu (12–15).

The gut–lung axis involves the generation of gut microbiota-derived metabolites within the colon, their distribution *in vivo* after crossing the intestinal mucosa, and their local exposure in the lung. Some molecules enter the portal vein after traversing the intestinal mucosa, merge into the systemic circulation after hepatic metabolic processing, and can reach lung tissues via the pulmonary circulation; other molecules mainly remain in the gut and exert local effects (16, 17). In addition, microaspiration of gastric contents can directly deliver specific molecules into the lumen of the lower respiratory tract (18). In COPD, changes in colonic substrate fermentation and the composition of related metabolites can continuously affect the state of pulmonary immune cells and airway structural cells through the gut–lung axis.

After entering the colon, complex carbohydrates serve as substrates for microbial fermentation and influence the composition of downstream metabolites. In the heterogeneous gut microecological environment of patients with COPD (19, 20), polysaccharide molecules in the diet that are not completely digested and absorbed by the host, such as fermentable dietary fiber, inulin-type fructans, and natural polysaccharides, can be degraded locally in the colon by bacterial enzymatic systems. The physicochemical characteristics of polysaccharide substrates, including monosaccharide composition, glycosidic linkage type, degree of branching, and degree of polymerization, can affect the recognition and degradation efficiency of microbial carbohydrate-active enzymes (CAZymes) (21–23). Differences in the rate of enzymatic degradation further alter the sites at which carbohydrate fermentation occurs within the colon: components with a low degree of polymerization are mostly utilized rapidly in the proximal colon, whereas structurally more complex polysaccharides, due to their slower enzymatic degradation, can undergo fermentation extending to the distal colon (21, 24, 25). Spatial changes in carbohydrate abundance within the colon further alter the production ratio of short-chain fatty acids (SCFAs) and tryptophan metabolites, with additional influences on bile acid-related signaling and protein fermentation products (24–28).

Some metabolites generated in the colon can cross the intestinal mucosa, enter the systemic circulation, and reach the lung. Among these metabolites, SCFAs and tryptophan metabolites are the principal candidates for systemic gut-to-lung signaling, whereas bile acids in COPD are more closely associated with microaspiration from gastroesophageal reflux and local microecological alterations in the lower respiratory tract. Gut-derived molecules that reach the lung can act on immune cells and airway structural cells and affect neutrophil recruitment, alveolar macrophage function, the Treg/Th17 balance, and the status of the airway epithelial barrier (29–37).

**Figure 1:**
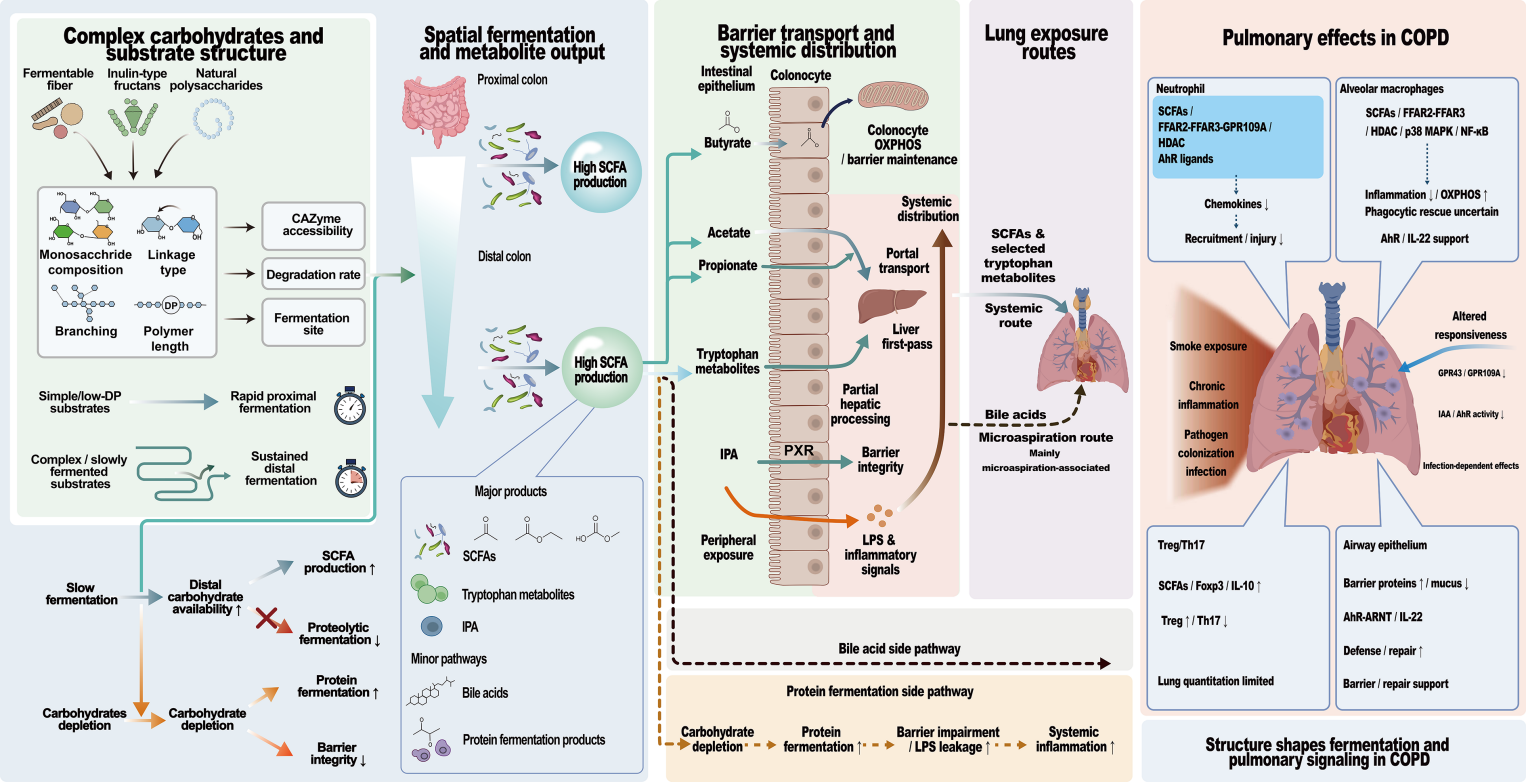
Overall mechanistic framework by which complex carbohydrates influence immune imbalance in COPD via the gut–lung axis. Complex carbohydrate structure shapes colonic fermentation kinetics and metabolite generation. Acetate, propionate, and selected tryptophan metabolites can reach the lung through the systemic circulation, whereas butyrate is mainly utilized locally in the gut. In COPD, bile acids are more closely associated with microaspiration-related lower-airway exposure. Gut-derived metabolites may influence neutrophil recruitment, alveolar macrophage inflammatory status, Treg/Th17 balance, and airway epithelial barrier integrity.

This review focuses on how structural differences in complex carbohydrates may influence immune imbalance in COPD by shaping colonic fermentation, metabolite generation, and downstream pulmonary responses through the gut–lung axis. Particular emphasis is placed on SCFAs and tryptophan metabolites as the main candidate effectors under current evidence, whereas bile acids are discussed more cautiously as a context-dependent pathway. The overall relationship by which complex carbohydrates influence immune imbalance in COPD via the gut–lung axis is shown in Figure 1.

## 2 Structural characteristics of colonic available substrates and colonic fermentation kinetics

Complex carbohydrate polymers that are not completely digested and absorbed by the host in the upper gastrointestinal tract, referred to as microbiota-accessible carbohydrates (MACs), enter the colon and constitute the core carbon source for gut microbiota metabolism (38, 39). The physicochemical characteristics of polysaccharide molecules, including monosaccharide composition, glycosidic linkage type, degree of branching, and degree of polymerization, affect the binding efficiency between microbial carbohydrate-active enzymes and polysaccharide substrates. Differences in enzymatic binding efficiency can cause spatial heterogeneity in the site and duration of polysaccharide degradation reactions within the colonic lumen (38–40). In addition, alterations in host intestinal transit time and local physicochemical conditions such as pH jointly influence the cleavage of structurally identical polysaccharide substrates in the colonic lumen into different combinations of initial metabolites (24, 38, 40, 41).

Along the anatomical axis of the intestine, the abundance of fermentable carbohydrates and the intensity of saccharolytic fermentation in the colonic lumen exhibit a spatial gradient that decreases from the proximal to the distal colon (25, 42). The proximal colonic lumen is enriched with a large amount of undegraded polysaccharide substrates, supporting active bacterial saccharolytic fermentation, generating high concentrations of SCFAs, and promoting a reduction in local pH (43). As luminal contents are propelled distally by peristalsis, readily accessible polysaccharide carbon sources are gradually depleted, and the intensity of saccharolytic fermentation correspondingly declines. Carbon source depletion drives the distal colonic microbiota to shift toward the degradation of proteins and amino acids, thereby increasing the production of nitrogen- and sulfur-containing metabolites such as branched-chain fatty acids, ammonia, and sulfides. Persistent carbon source depletion in the distal colon is likely to cause insufficient production of protective butyrate, weaken the oxidative phosphorylation-based energy metabolism of colonic epithelial cells, reduce the integrity of the mucosal barrier, and increase the probability that pro-inflammatory molecules such as lipopolysaccharide cross the epithelial structure (25, 42, 44). The rate of enzymatic degradation of polysaccharide substrates in the proximal colon participates in regulating the relative proportion of saccharolytic fermentation and proteolytic fermentation in the distal colonic lumen by determining the total amount of residual carbon substrate transported distally with the intestinal contents.

**Figure 2:**
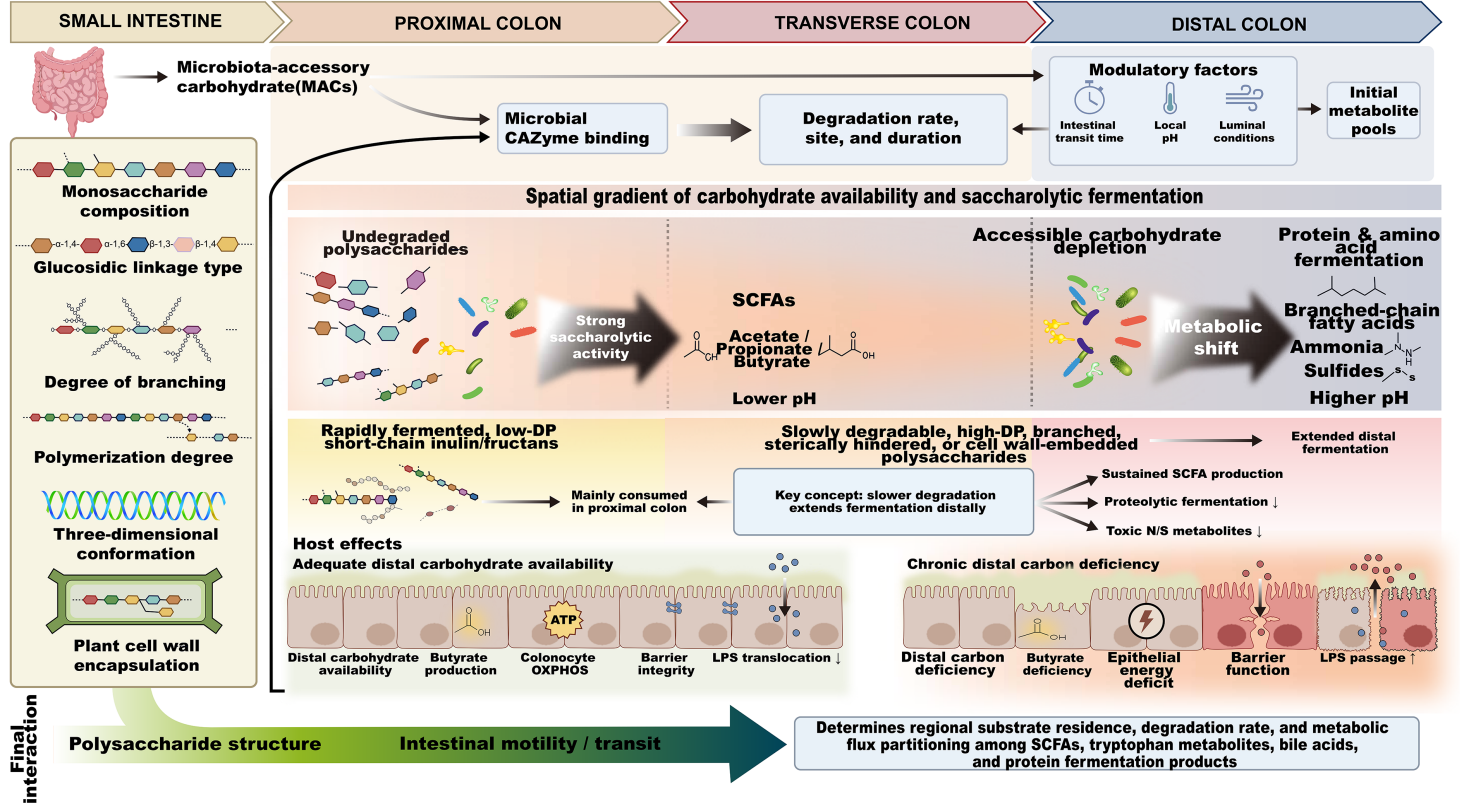
Effects of complex carbohydrate structural characteristics on the spatiotemporal kinetics of colonic fermentation. The structural characteristics of microbiota-accessible carbohydrates (MACs) determine their degradation rate, fermentation site, and fermentation duration in the colon. Structurally simple substrates are mainly utilized in the proximal colon, whereas more complex substrates can extend saccharolytic fermentation toward the distal colon. Sustained distal carbohydrate availability supports distal SCFA production and reduces proteolytic fermentation.

The more complex the spatial structure of a polysaccharide, the slower its enzymatic degradation in the colon is usually. This slow biochemical degradation process allows a large amount of uncleaved carbohydrate substrate to be propelled to the distal colon with colonic peristalsis, thereby extending the site of saccharolytic fermentation toward distal anatomical regions. Taking inulin-type fructans composed of linear fructose chains as an example, short-chain components with a low degree of polymerization are mostly rapidly utilized by bacteria in the proximal colon, whereas long-chain components with a high degree of polymerization undergo enzymatic degradation more slowly, and their fermentation can continue into the distal colon (24, 45). In contrast, natural polysaccharides with a high degree of branching, complex spatial conformation, or sequestration within the plant cell wall matrix reduce the probability of binding between gut bacterial degradative enzyme systems and the substrate through steric hindrance, thereby delaying glycosidic bond cleavage and the release of monosaccharide breakdown products (39, 41, 46). Residual complex polysaccharides transported with the intestinal contents to the distal colon provide a sustained carbohydrate substrate for the local microbiota in the distal region, which helps support the continuous synthesis of SCFAs in the distal colon. An adequate distal carbon supply promotes the preferential activation of local bacterial saccharolytic metabolic pathways, reduces bacterial degradation and consumption of proteins and amino acids, and decreases the accumulation of toxic products of proteolytic fermentation in the distal colonic lumen. The effects of different substrate structures on the spatiotemporal kinetics of colonic fermentation are shown in Figure 2. The corresponding relationships between different substrate structures and fermentation outcomes are shown in Table 1.

**Table 1:** Correspondence between substrate structural characteristics and fermentation outcomes.

Substrate structural characteristic	Representative substrate	Enzymatic degradation/fermentation kinetic characteristic	Main colonic distribution	Main metabolic outcome	Mechanistic implication for the distal colon
Low degree of polymerization, simple structure	Short-chain inulin-type fructans, structurally simple carbohydrates	Rapid enzymatic degradation, rapidly depleted in the proximal colon	Predominantly proximal	High proximal SCFA production	Limited distal saccharolytic support
High degree of polymerization, long-chain	Long-chain inulin-type fructans	Slow enzymatic degradation, delayed fermentation	Can extend to the distal colon	Prolonged duration of saccharolytic fermentation	Distal SCFA maintenance
High degree of branching, complex spatial conformation	Highly branched natural polysaccharides	Reduced probability of enzymatic binding, delayed cleavage	More likely to be retained to the distal colon	Enhanced distal saccharolytic fermentation	Reduced proteolytic compensation
Embedded within the plant cell wall	Embedded natural polysaccharides	Low accessibility, slower release	Greater retention in the distal colon	Sustained carbon supply	Reduced accumulation of proteolytic toxic metabolites

The spatial structure of dietary polysaccharides and the intestinal peristaltic transport mechanism, respectively, affect the local rate of enzymatic substrate degradation and the residence time within specific intestinal segments, thereby altering the distribution and consumption rate of carbohydrates across different regions of the colon. This spatial variation in substrate distribution and concentration provides the initial reaction conditions for microbial metabolism at different anatomical sites within the colonic lumen. Changes in local carbon source abundance and acid–base status may further shift the relative metabolic output of microbial fermentation, thereby influencing the balance between saccharolytic and proteolytic processes.

At present, available evidence more clearly supports the view that structural differences in complex carbohydrates can alter fermentation kinetics and spatial substrate distribution in the colon. However, in human COPD, it remains unclear to what quantitative extent these structural differences translate into changes in systemic or pulmonary exposure to SCFAs and tryptophan metabolites. Direct evidence from structurally defined interventions and synchronized metabolite quantification across relevant biological compartments is still limited.

## 3 Generation of gut-derived metabolites driven by colonic fermentation

Gut microbiota can generate SCFAs, tryptophan metabolites, secondary bile acids, and protein fermentation products in the colonic lumen through multiple parallel biochemical pathways (47, 48). Changes in carbohydrate abundance and local pH within different regions of the colon further affect the production ratio of these gut-derived metabolites within specific intestinal luminal compartments (26, 42, 49–51).

### 3.1 Multistep generation of SCFAs and interbacterial metabolite transfer

The physicochemical characteristics of polysaccharide molecules, such as glycosidic linkage type and complex spatial conformation, alter the binding efficiency between degradative enzymes and substrates, thereby affecting the proportion of oligosaccharide and monosaccharide fragments generated during the initial cleavage reactions (52). The generation of specific structural fragments promotes their preferential uptake and utilization by primary degraders equipped with the corresponding metabolic enzyme systems, converting them into primary fermentation products such as acetate, lactate, and succinate (53). For example, primary utilizers such as Bifidobacterium mainly produce acetate and lactate after cleaving specific oligosaccharide fragments (54).

Acetate, lactate, and succinate accumulated in the local intestinal lumen subsequently enter downstream bacterial fermentation branches and participate in the synthesis of secondary products. Butyrate-producing bacteria such as *Faecalibacterium prausnitzii* can take up environmental acetate to synthesize butyrate; lactate can be converted into propionate via the acrylate pathway, and can also be co-converted into butyrate together with free acetate in the metabolic branches of some butyrate-producing bacteria; succinate is mainly converted into propionate (55, 56). The generation of secondary products such as butyrate is highly dependent on the continuous transfer of intermediate metabolites among different bacterial strains. The initial physicochemical characteristics of polysaccharide substrates affect the transfer sequence and synthetic efficiency of intermediate metabolites among multiple strains by altering the types of early degradation fragments. For example, after the microbiota cleaves inulin-type fructans, the released intermediate products mainly flow into the butyrate synthesis pathway; in contrast, during the degradation of pectic polysaccharides rich in uronic acids, the intermediate products mainly enter the acetate and propionate synthesis pathways (57–59).

SCFAs generated in the colonic lumen cross the intestinal barrier through two pathways. Undissociated molecules pass through the apical membrane by free diffusion, whereas dissociated anions enter cells via monocarboxylate transporters such as MCT1/4 and SMCT1/2 (60). Butyrate entering the cell is preferentially directed into the mitochondrial oxidative phosphorylation pathway and serves as a core energy substrate for epithelial cells (61). Adequate butyrate oxidative metabolism helps upregulate tight junction protein expression and promote mucus secretion, thereby maintaining mucosal integrity. Acetate and propionate that are not consumed locally pass through the basolateral membrane and enter the portal vein. When butyrate supply is reduced or its oxidative utilization is impaired, paracellular permeability of the intestinal mucosa increases, promoting the passage of pro-inflammatory molecules such as luminal LPS across the mucosal intercellular space into the lamina propria. LPS entering the lamina propria then flows into the systemic circulation through the microcirculation, increasing the levels of pro-inflammatory factors in the peripheral bloodstream (62, 63).

Acetate and propionate entering the portal vein reach the liver with the blood flow and undergo first-pass clearance and metabolic modification. Propionate mainly participates in multienzyme-catalyzed reactions within the liver; acetate that is not cleared by the liver enters the peripheral systemic circulation through the venous blood (60, 64, 65). Because butyrate is extensively consumed by oxidative metabolism in the intestinal epithelium, and propionate undergoes highly efficient metabolic clearance in the liver, SCFAs in the peripheral blood exhibit a concentration gradient dominated by acetate, followed by progressively lower concentrations of propionate and butyrate (60, 64, 66). Free SCFAs entering the peripheral circulation are distributed by the bloodstream to distal organs such as the lung, where they bind to specific receptors on the surface of local target cells. However, these concentration gradients in peripheral blood do not necessarily reflect the bioactive concentrations of SCFAs in the pulmonary microenvironment of COPD.

### 3.2 Metabolic partitioning of tryptophan between the host and gut microbiota and ligand transformation

A small amount of dietary tryptophan that is not completely absorbed and reaches the colon simultaneously enters two types of processes: host uptake and microbial metabolism (49, 67). One portion of free tryptophan crosses the intestinal mucosa and is taken up by host cells, where it enters the kynurenine degradation pathway under the catalysis of IDO1 in local immune and mucosal cells (68, 69). Another portion of tryptophan retained in the intestinal lumen is converted by different microbial communities into distinct metabolites (70, 71). Bacteria carrying the tnaA gene mainly cleave tryptophan to produce indole; specific bacterial communities can also generate metabolites such as indole-3-propionic acid (IPA), indole-3-lactic acid (ILA), and indole-3-acetic acid (IAA) (72–74).

The abundance of fermentable carbohydrates in the colonic lumen affects the consumption rate of free tryptophan by altering local pH and the concentrations of immune factors (26, 49, 75). When carbohydrate supply is sufficient, acid production from saccharolytic fermentation promotes a decrease in luminal pH (49, 76). The lowered pH suppresses the enzymatic activity of some indole-producing bacteria, driving more tryptophan into the synthetic branches of ILA and IPA (26, 49). As fermentable carbohydrates become depleted, the local microbiota shifts to protein degradation (25, 77). The continuous accumulation of nitrogen- and sulfur-containing metabolites reduces the integrity of the intestinal mucosal barrier and increases the release of local pro-inflammatory factors (77–80). Elevated IFN-*γ* induces local cells to upregulate IDO1 expression, thereby accelerating the degradation of tryptophan to kynurenine within host cells (81–83).

Various tryptophan metabolites generated locally in the colon bind to host target receptors according to their specific chemical structures and undergo spatial transfer across the barrier. Microbiota-derived IPA mainly remains localized in the gut, enters cells, and binds to the pregnane X receptor (PXR), thereby participating in the maintenance of mucosal barrier function (84, 85). In contrast, molecules such as ILA, IAA, and indole not only bind locally to the aryl hydrocarbon receptor (AhR), but also partly cross the intestinal barrier and enter the portal venous blood flow. Portal venous blood transports gut-derived molecules to the liver. Gut-derived indole is converted in the liver into derivative forms such as indoxyl sulfate under the catalysis of cytochrome P450 enzymes and sulfotransferases. Sulfation modifies the water solubility and plasma protein-binding status of metabolite molecules, thereby affecting the distribution of free molecules to distal tissues (86–88). ILA and IAA molecules entering the systemic circulation through the hepatic veins are further affected by clearance through systemic excretory systems such as the kidney. The transintestinal input, hepatic processing level, and excretory clearance efficiency jointly determine the final plasma concentration of free molecules in the systemic circulation (74, 89, 90).

The systemic circulation transports free gut-derived ligand molecules to distal organs such as the lung. Molecules reaching lung tissue enter target cells and bind to the intracellular AhR transcription factor (89, 90). The downstream transcriptional changes induced by receptor activation are jointly influenced by the concentrations of specific inflammatory factors in the pulmonary microenvironment, the pathological status of the disease, and the characteristics of the target cells.

### 3.3 Microbial modification of bile acids and their context-dependent relevance in COPD

The human liver synthesizes primary bile acids such as cholic acid (CA) and chenodeoxycholic acid (CDCA), which are secreted into bile after biochemical conjugation in hepatocytes (91). A small fraction of these bile acids enters the colonic lumen, where bacterial deconjugation and secondary transformation reactions generate free primary and secondary bile acids (92–95). The abundance of carbohydrates in the colonic lumen can influence this transformation environment by altering local acid–base conditions. When carbohydrate supply is sufficient, acid production from saccharolytic fermentation lowers luminal pH and may suppress part of the secondary bile acid conversion process; as carbon sources become depleted, luminal pH rises and the efficiency of secondary transformation correspondingly increases (94, 96–98).

In COPD, however, the pulmonary relevance of bile acids appears to differ from that of SCFAs and tryptophan metabolites. Current evidence does not support bile acids as a dominant systemic colon-to-lung signaling class. Instead, their presence in the lower respiratory tract is more likely related to microaspiration from gastroesophageal reflux than to delivery through the systemic circulation after colonic fermentation (32, 99, 100). Bile acids in the local airway environment may mainly participate in altering the bacterial burden and microecological composition of the lower respiratory tract, whereas direct evidence that they function as colon-derived ligands driving immune transcriptional changes in the COPD lung remains limited.

### 3.4 Biochemical shift of the gut microbiota toward protein fermentation pathways under carbon source limitation

As intestinal contents progress to the distal colon, the abundance of fermentable carbohydrates decreases markedly (25, 101). Carbon source depletion drives some bacteria to shift toward the degradation of dietary and host-derived proteins (102, 103). Bacteria with proteolytic activity secrete proteases that cleave macromolecules into short peptides and free amino acids (102, 104). The amino acids subsequently undergo reactions such as transamination and deamination, generating metabolites including branched-chain fatty acids, ammonia, hydrogen sulfide, and phenolic compounds (105–107). At the same time, urea hydrolysis further increases the local concentrations of ammonia and ammonium ions (108, 109).

Continuously accumulated small molecules such as ammonia and hydrogen sulfide come into contact with local mucosal epithelial cells. Hydrogen sulfide reduces the electron transfer efficiency of mitochondrial respiratory chain complex IV, whereas high concentrations of ammonia interfere with mitochondrial oxygen consumption (77, 108). The decline in mitochondrial oxidative phosphorylation efficiency and ATP production reduces the cellular oxidative utilization of butyrate and alters mucosal barrier permeability (77, 110). Under conditions of elevated local pro-inflammatory factors, increased extracellular succinate binds to the SUCNR1 receptor on the surface of macrophages and dendritic cells, thereby increasing monocyte recruitment to the intestinal mucosa (111–114).

After local barrier permeability increases, aromatic metabolites such as phenol and p-cresol enter the portal vein, reach the liver through the bloodstream for biotransformation, and partially enter the systemic circulation (106, 115, 116). Unlike molecules such as SCFAs, there is currently no clear evidence that protein degradation products entering the systemic circulation directly bind to receptors in the distal lung (117–120).

At present, there is still a lack of quantitative evidence that protein degradation products generated in the colonic lumen directly bind to pulmonary target cells. In contrast, SCFAs and some tryptophan metabolites can cross the intestinal mucosa and be distributed to the lung through the systemic circulation, whereas bile acids in COPD are more closely associated with microaspiration from gastroesophageal reflux. The generation, systemic distribution characteristics, and pulmonary sites of action of the major gut-derived metabolites are detailed in Table 2.

**Table 2:** Generation, transport, and pulmonary actions of major gut-derived metabolites.

Metabolite category	Main generation mechanism	Systemic distribution characteristics	Mode of action in the lung	Mechanistic classification
SCFAs (acetate, propionate, butyrate)	Generated through multistep microbial fermentation of polysaccharides; acetate is a primary product, whereas propionate and butyrate depend on intermediate metabolite transfer and cooperative microbial conversion	Acetate has the highest concentration in peripheral blood, followed by propionate, with butyrate the lowest; butyrate is mainly oxidized and utilized locally in the intestinal epithelium	Enter the lung via the systemic circulation, bind to receptors on the surface of local target cells, and regulate inflammation- and barrier-related responses	Principal effector molecules
Tryptophan metabolites such as IAA/ILA/indole	Generated through microbial metabolism of tryptophan; different microbial communities determine the composition of branched products such as IAA, ILA, and indole	Partly act locally and partly enter the liver via the portal vein and then the systemic circulation; influenced by hepatic processing and renal clearance	After entering the lung, they enter target cells and bind to AhR, thereby regulating downstream transcription	Principal effector molecules
IPA	Product of the reductive pathway in specific Clostridium-like bacterial communities	Mainly remains localized in the gut	Mainly maintains the intestinal barrier through PXR	Local protective molecule
Bile acids	Secondary bile acids are formed from primary bile acids through microbial deconjugation and 7α-dehydroxylation	Dominated by enterohepatic circulation; pulmonary relevance in COPD is more likely linked to microaspiration than to systemic delivery	Exposure in the lower respiratory tract is mainly associated with microaspiration, with greater effects on the local microecology; direct immunoregulatory evidence remains limited	Context-dependent bypass signal
Protein fermentation products	Generated from protein/amino acid degradation after carbon source depletion	Partly enter the systemic circulation, but mainly act first on the local intestinal mucosa	Mainly affect the lung indirectly through barrier disruption and systemic inflammation	Indirect detrimental factors

## 4 Classification-based regulation of pulmonary immune features in COPD by gut-derived metabolites

**Figure 3:**
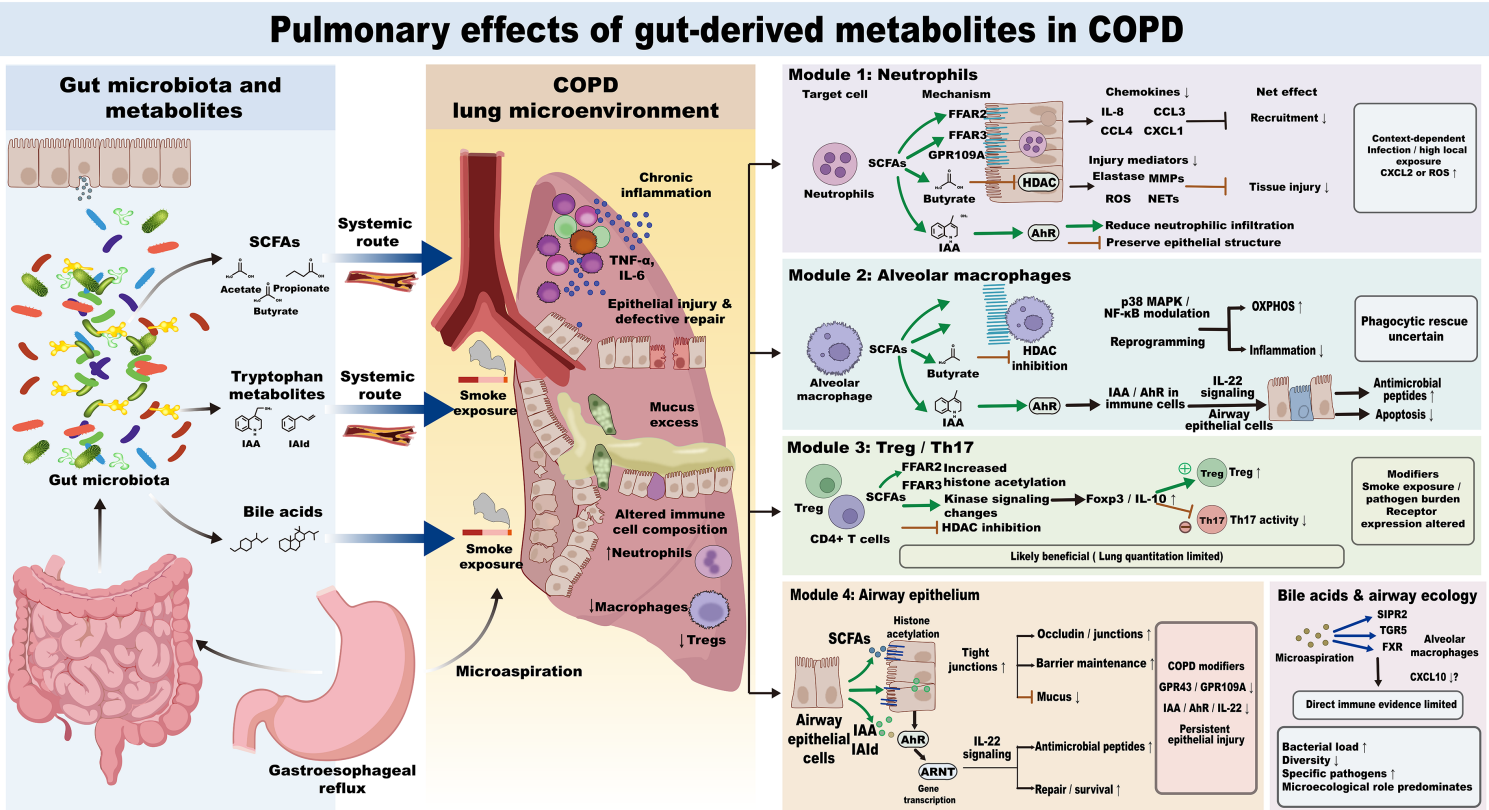
Regulatory patterns of gut-derived metabolites on key pulmonary pathological nodes in COPD. SCFAs and tryptophan metabolites mainly reach the lung through the systemic circulation, whereas bile acids in COPD are more likely to enter the lower respiratory tract through microaspiration. SCFAs may regulate neutrophil recruitment, alveolar macrophage inflammatory status, Treg/Th17 balance, and airway epithelial barrier function, whereas tryptophan metabolites mainly support mucosal defense and epithelial repair. These effects may vary with disease stage, infection status, smoking exposure, and local receptor responsiveness.

After reaching the pulmonary microenvironment, gut-derived metabolites can act on different target cells and influence cellular states through corresponding receptors or intracellular pathways. Under the pathological conditions of COPD, these metabolites mainly participate in regulating four specific cellular activities: local recruitment of neutrophils, phagocytic clearance function of alveolar macrophages, the distribution ratio of Treg and Th17 lymphocytes, and the barrier integrity of the airway epithelium. SCFAs and some tryptophan metabolites directly participate in signaling involved in local immune cell activation and epithelial tissue repair; bile acid molecules are mainly exposed to the lower respiratory tract in association with microaspiration from gastroesophageal reflux and have limited direct signaling effects on local pro-inflammatory mechanisms in COPD. At present, however, the strength of evidence is not uniform across these metabolite classes, and the ultimate pulmonary effects of individual metabolites are likely to depend on ligand concentration, disease stage, infection status, and local receptor responsiveness. The regulatory patterns of gut-derived metabolites on key pulmonary pathological nodes in COPD are shown in Figure 3, and the related receptor-binding effects and limiting conditions are shown in Table 3.

**Table 3:** Relationships between key pathological nodes in COPD and regulation by gut-derived metabolites.

Key pathological node	Gut-derived metabolite	Key receptor/pathway	Main effect	Pathological modifiers/evidence boundary
Aberrant neutrophil recruitment	SCFAs	FFAR2, FFAR3, GPR109A, HDAC	IL-8/CCL3/CCL4/CXCL1 ↓; neutrophil recruitment ↓; proteases/MMPs/ROS/NETs ↓; tissue injury ↓	Context-dependent; in specific infection models, butyrate may cause CXCL2 ↑ and neutrophil recruitment ↑
Aberrant neutrophil recruitment	Tryptophan metabolites such as IAA	AhR	Neutrophil infiltration ↓; epithelial integrity ↑	Context-dependent; high concentrations of IAA may cause ROS ↑ and tissue injury ↑ in acute bacterial pneumonia models
Impaired alveolar macrophage clearance function	SCFAs	FFAR2, FFAR3, HDAC, p38 MAPK, NF-κB	Oxidative phosphorylation proportion ↑; pro-inflammatory cytokine secretion ↓	Evidence more strongly supports modulation of inflammatory status than direct improvement of phagocytic clearance in COPD
Impaired alveolar macrophage clearance function	Tryptophan metabolites such as IAA	AhR / IL-22-related signaling	IL-22-related signaling ↑; antimicrobial peptide expression ↑; epithelial apoptosis ↓	In COPD models with decreased IAA, AhR activity ↓, IL-22-related signaling ↓, and barrier injury ↑
Impaired alveolar macrophage clearance function	Specific secondary bile acids (such as isoLCA)	S1PR2, TGR5, FXR	CXCL10 ↓; local inflammatory responses may be altered	Evidence remains insufficient that these molecules directly improve macrophage function in COPD
Treg/Th17 imbalance	SCFAs (butyrate, some propionate)	FFAR2, FFAR3, HDAC	Foxp3/IL-10 ↑; Treg differentiation ↑; Th17 activation ↓	Mechanistically plausible, but in vivo quantitative evidence in COPD remains insufficient; may be modified by smoking exposure and pathogen colonization
Airway epithelial barrier injury and impaired repair	SCFAs	Epithelial cell surface receptors, histone acetylation	occludin/ZO-1 ↑; excessive mucus secretion ↓; barrier integrity ↑	In COPD, GPR43/GPR109A ↓, and responsiveness to SCFAs may be reduced
Airway epithelial barrier injury and impaired repair	Tryptophan metabolites such as IAA and IAld	AhR, ARNT, IL-22-related signaling	IL-22-related signaling ↑; antimicrobial peptides ↑; epithelial repair ↑; epithelial apoptosis ↓	In COPD models with decreased IAA, AhR activation ↓, IL-22-related signaling ↓, and persistent barrier injury ↑
Lower respiratory tract microecological alterations	Bile acids	S1PR2, TGR5, FXR	Total bacterial burden ↑; community diversity ↓; proliferation of specific pathogens ↑	Mainly associated with microaspiration; current evidence supports a context-dependent microecological role rather than a principal gut-derived immunoregulatory mechanism in COPD

### 4.1 Gut-derived regulation of aberrant neutrophil recruitment

Aberrant accumulation of neutrophils in the local airway is often accompanied by the extracellular release of elastase, matrix metalloproteinases (MMPs), reactive oxygen species (ROS), and neutrophil extracellular traps (NETs), thereby contributing to degradation of the alveolar matrix and destruction of the small airway wall (121). SCFAs entering the pulmonary microenvironment can bind to transmembrane receptors on the surface of target cells. Among them, acetate and propionate mainly bind to free fatty acid receptor 2 (FFAR2) and free fatty acid receptor 3 (FFAR3); butyrate mainly binds to G protein-coupled receptor 109A (GPR109A), and can also enter target cells and inhibit the catalytic activity of histone deacetylases (HDACs), thereby altering gene transcription within target cells (122).

In some noninfectious airway inflammation models, exposure to SCFAs can reduce the secretion of chemokines such as IL-8, CCL3, CCL4, or CXCL1 by target cells, and decrease the migration and accumulation of neutrophils in the local lung environment (123–125). As local neutrophil accumulation decreases, the total local release of proteases, ROS, and NETs in the airway correspondingly declines, which may help alleviate tissue injury in the alveolar septa and small airway walls (121). In contrast, in specific pathogenic infection models, butyrate exposure can upregulate CXCL2 expression in pulmonary macrophages and may increase neutrophil migration and accumulation in the lung (123).

In the tryptophan metabolic pathway, ligands such as indole-3-acetic acid (IAA), after entering local target cells, can activate the AhR pathway. In COPD-related chronic inflammation models, exposure to such AhR ligands can attenuate local neutrophilic infiltration in the airway and help maintain the integrity of the airway epithelial structure (37). However, in specific acute bacterial pneumonia models, exposure to high concentrations of IAA can promote the extracellular release of ROS by neutrophils, thereby potentially aggravating pathological injury to alveolar tissue (89).

The infection status of the local airway microenvironment and ligand concentration jointly influence the ultimate regulatory effects of metabolite molecules on neutrophils. In a noninfectious airway inflammatory environment, SCFAs and some AhR ligands tend to reduce local neutrophil recruitment; however, in the presence of certain acute infections, exposure to some metabolites may increase local neutrophil accumulation or exacerbate tissue injury. These findings indicate that the effects of gut-derived metabolites on neutrophil recruitment in COPD should be interpreted as context-dependent rather than directionally fixed.

### 4.2 Gut-derived regulation of impaired alveolar macrophage clearance function

Alveolar macrophages phagocytose and clear local airway pathogens and apoptotic cells. In the pathological environment of COPD, these cells commonly exhibit increased secretion of pro-inflammatory factors, reduced phagocytic clearance capacity, and alterations in intracellular metabolic pathways.

SCFAs entering the pulmonary microenvironment bind to transmembrane receptors on the surface of alveolar macrophages, such as FFAR2 and FFAR3; among them, butyrate can also enter the cells and inhibit the catalytic activity of HDACs. Activation of transmembrane receptors and reduction of intracellular enzymatic activity alter the phosphorylation status of p38 mitogen-activated protein kinase (p38 MAPK) and nuclear factor-κB (NF-κB) in the cytoplasm, thereby regulating gene transcription within the target cells (63, 122, 126, 127). Changes in transcriptional status promote macrophages to increase the metabolic proportion of the oxidative phosphorylation pathway and reduce the secretion of some pro-inflammatory cytokines (123, 128).

In specific early-stage pathogen infection models, the increase in the intracellular proportion of oxidative phosphorylation in macrophages did not synchronously increase the number of local bacteria phagocytosed by these cells (123, 128). In the local COPD-related environment, exposure to SCFAs tends to reduce the release of pro-inflammatory factors by alveolar macrophages, but may not directly increase their phagocytic and clearance capacity toward pathogens. Thus, current evidence more strongly supports an effect of SCFAs on macrophage inflammatory status and metabolic programming than on direct restoration of phagocytic clearance function in COPD.

In the tryptophan metabolic pathway, ligand molecules such as IAA can activate the AhR pathway in local immune cells. Following activation of the AhR pathway, IL-22-related signaling is enhanced and influences the local mucosal defense status through communication between immune cells and airway epithelial cells. Enhanced IL-22 signaling can act on adjacent airway epithelial cells, inducing antimicrobial peptide expression and reducing apoptosis (37, 129–131). In some COPD models, decreased local airway IAA concentration is accompanied by reduced AhR pathway activity and weakened IL-22-related signaling, which may aggravate airway epithelial barrier injury (37).

### 4.3 Gut-derived regulation of Treg/Th17 imbalance

Treg/Th17 imbalance is observed in COPD, and the local airway environment is also commonly accompanied by a reduction in regulatory T cells (Treg) and increased activation of T helper 17 (Th17) cells. SCFAs may participate in the regulation of this imbalance by affecting the local immune environment in the lung or by influencing the differentiation status of peripheral lymphocytes. Relevant SCFA molecules can bind to FFAR2 and FFAR3 on the surface of target cells and alter the intracellular phosphorylation status of kinases; meanwhile, butyrate and some propionate molecules can also enter cells and inhibit the catalytic activity of HDACs (122).

Alterations in intracellular kinase status and the reduction in HDAC activity promote the maintenance of a high level of histone acetylation in specific chromatin regions, thereby upregulating the mRNA transcription of genes such as forkhead box protein P3 (Foxp3) and interleukin-10 (IL-10) (125, 132). Increased synthesis of Foxp3 protein induces lymphocyte differentiation toward Treg and may suppress excessive local activation of Th17 cells and their cytokine secretion (36, 126).

At present, sufficient direct *in vivo* evidence is still lacking to quantify the absolute local concentrations and cellular effects of the above SCFA molecules in the lungs of patients with COPD. In addition, long-term cigarette smoke exposure and local proliferation of specific pathogens may alter the expression levels of receptors on local lymphocytes, thereby affecting the ultimate regulatory outcome of SCFAs on the distribution ratio of Treg and Th17 cells in the airway local environment. Therefore, current evidence supports the mechanistic plausibility of SCFA-mediated regulation of Treg/Th17 balance, but not yet a quantitatively resolved causal pathway in human COPD.

### 4.4 Gut-derived regulation of airway epithelial barrier injury and impaired repair

COPD is commonly accompanied by a reduction in the number of airway epithelial cells, disruption of intercellular tight junctions, and decreased secretion of antimicrobial peptides. SCFAs such as acetate, propionate, and butyrate, which reach the local airway environment through the systemic circulation, bind to transmembrane receptors on the surface of epithelial cells or enter the cells and alter the histone acetylation status of specific chromatin regions. The resulting changes in the transcriptional state of target cells upregulate the synthesis of occludin and zonula occludens-1 (ZO-1), thereby helping to maintain the mucosal barrier structure and possibly reducing excessive airway mucus secretion in some local microenvironments (128).

Free tryptophan metabolites, such as IAA and indole-3-aldehyde (IAld), can bind to the cytoplasmic AhR after entering local target cells. Ligand binding triggers AhR translocation into the nucleus, where it forms a complex with AhR nuclear translocator (ARNT), thereby regulating the transcription of downstream target genes (133). Following activation of the AhR pathway, IL-22-related signaling is enhanced and promotes mucosal defense and tissue repair through communication between local immune cells and epithelial cells. Enhanced IL-22 signaling can act on airway epithelial cells, induce the synthesis of antimicrobial peptides, and may promote the proliferation and repair of injured epithelial cells (29, 129, 130, 134).

In the pathological environment of COPD, long-term cigarette smoke exposure downregulates the expression of receptors such as GPR43 and GPR109A in lung tissue (135). The reduction in the number of transmembrane receptors decreases the binding rate of SCFAs to target cells and the subsequent transcriptional changes. At the same time, some COPD models show decreased local airway concentrations of ligands such as IAA (37). The reduction in IAA ligand concentration weakens the transcriptional activation of the AhR pathway and decreases IL-22-related signaling, accompanied by reduced synthesis of antimicrobial peptides and increased apoptosis of epithelial cells, which may promote the persistence of airway epithelial injury (37, 129, 130). These findings suggest that effective epithelial protection depends not only on metabolite availability, but also on the preservation of receptor expression and downstream signaling responsiveness in diseased lung tissue.

### 4.5 Lower respiratory tract exposure to bile acid molecules and local microecological alterations

Free bile acid molecules enter the lumen of the lower respiratory tract mainly in association with gastroesophageal microaspiration, rather than primarily through delivery by the systemic circulation after colonic fermentation (18, 131, 136). In some models, specific secondary bile acid molecules reaching the local airway environment, such as isolithocholic acid (isoLCA), can bind to sphingosine-1-phosphate receptor 2 (S1PR2) or the G protein-coupled receptor TGR5 on the surface of alveolar macrophages, or enter cells and bind to farnesoid X receptor (FXR), thereby potentially inhibiting the secretion of specific chemokines such as CXCL10 (32, 100, 137).

In the local airway environment of patients with COPD, the accumulation of free bile acid molecules is accompanied by an increased total bacterial burden, reduced community diversity, and proliferation of specific pathogens (18). At present, sufficient direct evidence is lacking to show that changes in airway bile acid concentrations directly drive abnormal immune secretion by pulmonary target cells or airway tissue injury in COPD. Bile acid molecules in the local airway environment may be more involved in altering the bacterial community structure of the pulmonary microecology, rather than directly acting as cross-organ ligands derived from colonic fermentation to drive abnormal activation of host immune cells. Accordingly, bile acids in COPD are better interpreted as a context-dependent airway exposure signal than as a principal gut-derived effector class equivalent to SCFAs or tryptophan metabolites.

## 5 Limitations and translational perspectives

Current studies on how complex carbohydrates influence immune imbalance in COPD via the gut–lung axis are derived mainly from ex vivo fermentation systems, cell-based experiments, or specific animal models. The extraction purity, processing methods, and physicochemical characteristics of the polysaccharide substrates used in different studies are not consistent, and dietary fibers or natural polysaccharides with the same name do not necessarily correspond to the same colonic available substrates across different experiments. In the actual gut microenvironment of patients with COPD, there is still a lack of direct human evidence as to whether polysaccharides with specific monosaccharide compositions, glycosidic linkage types, degrees of branching, and degrees of polymerization can stably extend to the distal colon and produce similar fermentation outcomes. SCFAs and tryptophan metabolites generated in the colon must still undergo intestinal mucosal transport, hepatic metabolism, and peripheral tissue consumption before entering the systemic circulation, and their free concentrations in peripheral blood may not reflect the actual molecular abundance in lung interstitial fluid or airway surface liquid. At present, there is still a lack of synchronous measurements from feces, peripheral blood, and lower respiratory tract samples obtained from the same subjects. In contrast, bile acids in COPD are more closely associated with gastroesophageal microaspiration, and the actual changes in their local concentrations and duration of persistence in the lower respiratory tract also remain unclear.

The cellular effects induced by gut-derived molecules reaching the local airway environment may not be consistent across different pathological conditions. Cigarette smoke exposure, pathogen colonization, and acute inflammation can alter the expression levels of receptors on the surface of airway cells, as well as intracellular kinase activity and transcriptional status. Therefore, the same concentration of SCFAs or tryptophan metabolites may correspond to different outcomes in neutrophil recruitment, macrophage function, Treg/Th17 balance, and epithelial repair at different stages of disease. Future studies may synchronously collect fecal, peripheral blood, and lower respiratory tract samples during both the stable phase and acute exacerbation phase of COPD, determine the concentration changes of SCFAs and major tryptophan metabolites at different sites, and combine this approach with stable isotope labeling techniques to trace the metabolic fate of structurally defined carbohydrates *in vivo*, thereby clarifying the colonic fermentation patterns corresponding to different substrate structures, the pulmonary exposure profiles of related metabolites, and their principal target cell types in the local airway environment of COPD.

From a translational perspective, future clinical nutrition studies should move beyond the broad use of the term “dietary fiber” and instead evaluate structurally defined complex carbohydrates with clear monosaccharide composition, glycosidic linkage type, degree of branching, and degree of polymerization. Intervention design should also specify dose, duration, background diet, and formulation, so that structure-dependent fermentation patterns and metabolite responses can be compared across studies. For functional product development, candidate substrates with better distal colonic delivery potential, batch consistency, and reproducible fermentation characteristics may have greater practical value for clinical nutrition and industrial application in COPD.

## 6 Conclusion

Complex carbohydrates may participate in the regulation of immune imbalance in COPD by influencing the process of colonic fermentation. The monosaccharide composition, glycosidic linkage type, degree of branching, and degree of polymerization of polysaccharides are likely to affect their degradation rate and fermentation site in the colon, thereby altering the production profile of SCFAs and tryptophan metabolites. SCFAs are the main candidate metabolites distributed to the lung via the systemic circulation and are linked to the regulation of aberrant neutrophil recruitment, alveolar macrophage inflammatory status, Treg/Th17 imbalance, and airway epithelial barrier integrity; selected tryptophan metabolites mainly influence mucosal defense and epithelial repair through AhR-related pathways. In COPD, bile acids are more closely associated with gastroesophageal microaspiration and local microecological alterations in the lower respiratory tract. Further studies are needed to clarify how structurally defined complex carbohydrates shape colonic fermentation patterns, metabolite distribution, and local pulmonary responses in COPD through synchronized quantitative measurements across the gut, blood, and lung and through human intervention studies.

## Glossary

AhR: aryl hydrocarbon receptor
ARNT: aryl hydrocarbon receptor nuclear translocator
ATP: adenosine triphosphate
CA: cholic acid
CAZymes: carbohydrate-active enzymes
CCL3: C-C motif chemokine ligand 3
CCL4: C-C motif chemokine ligand 4
CDCA: chenodeoxycholic acid
COPD: chronic obstructive pulmonary disease
CXCL1: C-X-C motif chemokine ligand 1
CXCL2: C-X-C motif chemokine ligand 2
CXCL10: C-X-C motif chemokine ligand 10
DCA: deoxycholic acid
FFAR2: free fatty acid receptor 2
FFAR3: free fatty acid receptor 3
Foxp3: forkhead box protein P3
FXR: farnesoid X receptor
GPR43: G protein-coupled receptor 43
GPR109A: G protein-coupled receptor 109A
HDAC: histone deacetylase
IAA: indole-3-acetic acid
IAld: indole-3-aldehyde
IDO1: indoleamine 2,3-dioxygenase 1
IFN-*γ*: interferon-gamma
ILA: indole-3-lactic acid
IL-8: interleukin-8
IL-10: interleukin-10
IL-22: interleukin-22
IPA: indole-3-propionic acid
isoLCA: isolithocholic acid
LCA: lithocholic acid
LPS: lipopolysaccharide
MACs: microbiota-accessible carbohydrates
MCT1/4: monocarboxylate transporter 1/4
MMPs: matrix metalloproteinases
NETs: neutrophil extracellular traps
NF-κB: nuclear factor kappa-B
p38 MAPK: p38 mitogen-activated protein kinase
PXR: pregnane X receptor
ROS: reactive oxygen species
SCFAs: short-chain fatty acids
SMCT1/2: sodium-coupled monocarboxylate transporter 1/2
S1PR2: sphingosine-1-phosphate receptor 2
SUCNR1: succinate receptor 1
TGR5: Takeda G protein-coupled receptor 5
Th17: T helper 17 cell
tnaA: tryptophanase gene A
Treg: regulatory T cell
ZO-1: zonula occludens-1
